# Sensitivity Analysis of Intensity-Modulated Plastic Optical Fiber Sensors for Effective Aging Detection in Rapeseed Transformer Oil

**DOI:** 10.3390/s23249796

**Published:** 2023-12-13

**Authors:** Ugochukwu Elele, Azam Nekahi, Arshad Arshad, Kate McAulay, Issouf Fofana

**Affiliations:** 1 School of Computing, Engineering and Built Environment, Glasgow Caledonian University, Glasgow G4 OBA, UK; ugochukwu.elele@gcu.ac.uk (U.E.); arshad.arshad@gcu.ac.uk (A.A.); kate.mcaulay@gcu.ac.uk (K.M.); 2 Department of Applied Sciences, Université du Québec à Chicoutimi, Saguenay, QC G7H 2B1, Canada

**Keywords:** degradation, online, transformer oil, refractive index, evanescent field

## Abstract

As the focus tilts toward online detection methodologies for transformer oil aging, bypassing challenges associated with traditional offline methods, such as sample contamination and misinterpretation, fiber optic sensors are gaining traction due to their compact nature, cost-effectiveness, and resilience to electromagnetic disturbances that are typical in high-voltage environments. This study delves into the sensitivity analysis of intensity-modulated plastic optical fiber sensors. The investigation encompasses key determinants such as the influence of optical source wavelengths, noise response dynamics, ramifications of varying sensing lengths, and repeatability assessments. Our findings highlight that elongating sensing length detrimentally affects both linearity response and repeatability, largely attributed to a diminished resistance to noise. Additionally, the choice of the optical source wavelength proved to be a critical variable in assessing sensor sensitivity.

## 1. Introduction

Transformers play an indispensable role in power delivery to end users. Their vital function, combined with their high cost and extensive maintenance, emphasizes their importance. Any malfunction within the transformer system can halt an entire power station, leading to significant economic consequences, potential threats to life, damage to equipment, and environmental implications [1]. Therefore, the continuous monitoring of the transformer’s state of health (SOH) is crucial. In oil-filled transformers, oil plays essential roles such as cooling, insulating, and arc suppression and acts as a primary indicator of the transformer’s operational SOH. Over time, due to factors like transformer loading and thermal stress, the oil deteriorates [2], producing aging by-products (ABPs) that compromise its physicochemical properties. These ABPs include gases, acids, and sludge [3,4,5]. Various offline methods, which encompass the chemical, electrical, physical, and spectroscopic techniques [3,6,7,8,9,10,11,12,13,14], are employed to characterize transformer oil (TO). Generally, the methods to detect transformer aging are categorized as intrusive or non-intrusive, destructive or non-destructive, and offline or online. Among these, non-destructive online techniques are the most advantageous but require the selection of an appropriate sensor [6].

Recent studies underscore the growing attention toward online aging sensors designed for TO detection, prominently featuring ABP gas sensors [15], cross-capacitance sensors [16,17], and fiber-optic sensors [11]. Fiber-optic sensors excel due to their insulating properties, resistance to electromagnetic interference, compactness, lightweight design, high sensitivity, broad bandwidth, adaptability in extreme conditions, resilience, and compatibility with remote and distributed sensing applications [11,18,19]. These sensors are particularly adept at monitoring physical parameters such as strain, temperature, and humidity. Optical fibers are categorically classified based on their material composition (e.g., plastic, glass, polymer, silicon), refractive index (RI) profiles, and light propagation modes. Notably, multimode fibers possess larger diameters facilitating multiple light propagation pathways, while single-mode fibers, with their narrower diameter, restrict the light to a single mode, eliminating modal dispersion. The sensitivity responses of these fibers are inherently influenced by their material selection, RI, and propagation mode. A study by [20] indicated that silicon fibers, when used for acidity monitoring, demonstrated enhanced stability and repeatability compared to polymer fibers. Conversely, polymer fibers were noted for their heightened sensitivity and superior flexibility [19,21].

An optical fiber comprises a core, cladding, and coating. The core, made of either glass or plastic, facilitates light transmission. The cladding, with a lower RI than the core, ensures that light reflects within the core using the principle of total internal reflection [19], minimizing scattering loss and protecting the core. The coating, typically plastic, offers environmental and mechanical protection for the fiber [19]. Optical fiber sensor instrumentation system has three primary elements: the optical transmitter, the sensing region, and the detector. A segment of the optical fiber is stripped to form the sensing area. The light sources can be light-emitting diodes (LEDs) or laser diodes. Optical fiber sensors fall into intrinsic or extrinsic categories [19,22]. Intrinsic sensors utilize a stripped section of the optical fiber itself as the sensor, directly interacting with the material being measured to modulate a light property. Extrinsic sensors, however, channel light to an external sensor and are less popular than intrinsic sensors. Intrinsic configurations measure changes in light wave parameters such as wavelength, phase, and intensity. Consequently, optical fiber sensors can be phase-, intensity-, wavelength-, or polarization-modulated [19]. Optical fiber sensors are increasingly utilized in diverse fields such as environmental surveillance, biomedical diagnosis and therapy, biochemical analysis, and the food industry [21,23,24]. More detailed reviews and applications of sensors based on changes in RI can be found in [1,21,25,26].

In high-voltage transformer applications, the adoption of plastic optical fiber sensors has been under-researched compared to other offline characterization methodologies. This study specifically delves into a sensitivity analysis of plastic optical fiber sensors within transformers utilizing rapeseed natural ester oil. Subsequent sections elucidate the fundamental principles underpinning the chosen sensor, outline the experimental materials and methodologies employed, analyze the sensitivity of pivotal variables within the instrumentation system, and provide a comprehensive discussion and conclusion of the most important findings. Furthermore, recommendations are presented for enhancing sensitivity in TO applications.

## 2. Principles of Intensity-Modulated Fiber Optic Instrumentation

When light transitions between different media, its velocity alters, leading to the phenomenon of refraction. Light propagates most rapidly in a vacuum compared to any other medium. The RI of a substance, such as water or TO, quantifies the variation in light’s velocity as it transitions from a vacuum (or air, for practical purposes) into that substance. This relationship can be mathematically represented as follows:

(1) $$n=\frac{V_{1}}{\;V_{2{\;}_{\;}}}$$

where n represents the RI of the material; $V_{1}$ represents the speed of light in a vacuum; and $V_{2}$ represents the speed of light in a material. The evanescent field absorption-based configuration is a widespread intrinsic sensing technique [27]. The parameter to be detected affects the RI of the uncladded portion, prevents optical waves from leaking into the fiber optic waveguide [27], and consequently diminishes some properties of the light wave reaching the fiber output.

This can be mathematically expressed as follows [27]:

(2) $$\mu =\frac{P_{clad}}{P_{clad{\;}_{\;}}+P_{core}}≅\frac{4}{3V}$$

 μ is the modal fractional power, $P_{clad}\;and\;P_{core}$ are the powers carried on the core and cladding, and V is the fiber’s normalized frequency. The normalized frequency is a unitless quantity expressed as follows:

(3) $$V=\frac{2\pi a}{\lambda }\sqrt{{n^{2}}_{core}-{n^{2}}_{clad}}$$

a is the fiber core radius, λ is the wavelength of the optical source, and $n_{core}\;and\;n_{clad}$ are the refractive indices of the core and cladding, respectively. With the constant source wavelength, core diameter, and core RI, $P_{clad}$ becomes proportionally related to the clad RI, $n_{clad}$ (combining Equations (2) and (3)). The uncladded region (sensing area) makes direct contact with the aging TO sample, and so $n_{clad}$ is replaced with $n_{oil}$, and $P_{clad}$ is replaced by $P_{oil}$, representing the power lost to the TO due to changes in the RI of the oil. This loss modulates the intensity at the end of the optical fiber and forms the basis for inferring the concentrations or purity of the oil in contact with the uncladded section of the optical fiber sensor, which is caused by evanescent field interaction with the oil and the RI of the oil [21,23,28,29].

Optical properties such as light scattering, absorption, reflection, and dispersion are influenced by the RI of the medium in which they travel. Consequently, the RI is a critical parameter in fiber optic instrumentation. [21,23]. Aging increases the RI of the TO and consequently increases the power lost to the TO. This power will not get to the fiber optic output, thus defining a sensing area (uncladded) that responds to the TO aging behavior. Optical fiber sensors have garnered significant traction in sectors such as environmental monitoring, biomedical diagnostics and treatments, biochemical material analysis, and the food industry [21,23].

## 3. Materials and Methods

### 3.1. Sample Aging and Standard

An exhaustive examination of the degradation trajectory of TO—from inception to culmination—necessitates a prolonged time period, ranging from fifteen to fifty years, and is dedicated to natural aging observation, data acquisition, and data analysis [30]. This timeframe often surpasses those used for conventional research periods. Hence, to deeply probe into the aging phenomenon and its effects on dielectric properties, there is an imperative need for a condensed, yet representative, replication of natural aging dynamics, colloquially referred to as accelerated aging techniques. These techniques introduce an amalgamation of electrical and environmental stresses that emulate real-world conditions, strictly in alignment with established standards. In this investigation, the aging procedure of TO adhered to the ASTM D1934-20 protocol [31]. This specific standard elucidates two distinct methodologies for inducing aging in TO: one is devoid of a metal catalyst (Procedure A) and the other incorporates a metal catalyst (Procedure B). Utilizing a metal catalyst, such as copper, increases the oxidation rate. This research employed Procedure B. To facilitate a nuanced exploration of the contributory roles of copper and paper in speeding up the aging process, the samples were systematically bifurcated into four categories: solely oil (samples 1–9), oil in conjunction with paper (65 g, dimensions—7.5 cm × 7 cm; samples 10–12), oil supplemented with copper (12 g/L concentration; samples 13–15), and an amalgamation of oil, paper, and copper (65 g paper, 7.5 cm × 7 cm in size, with a 12 g/L copper concentration; samples 16–18).

The apparatus includes the following:

1. An oven capable of maintaining a constant temperature of 115 °C (ASTM D1934-20);
2. Beakers;
3. 18 × 1000 mL Pyrex narrow-mouthed conical flask;
4. Natural ester TO (rapeseed);
5. Aging timer;
6. Distilled water;
7. Copper (9 g) and paper (65 g) catalysts.

**Figure 1:**
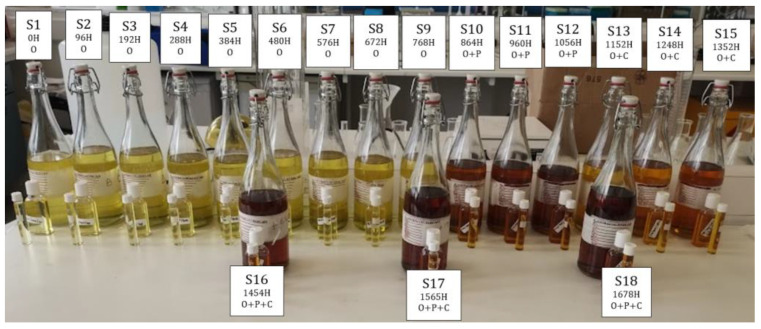
Samples 1–18 showing hours (H) of aging for oil (O), paper (P), and copper (C) combinations.

The procedural steps were as follows:

1. Sample containers were meticulously prepared and designated with labels from S1 through S18. Conforming to the guidelines stipulated in ASTM D1934-20 [31], a sampling duration extending a minimum of 96 h was strictly maintained.
2. A volume of 750 mL of pristine rapeseed oil was allocated to the container marked S1. In a similar manner, 750 mL of the identical oil specimen was allocated to each of seventeen (17) uncontaminated, narrow-mouthed conical flasks, culminating in a total of eighteen (18) samples with congruent mass. In adherence to ASTM D1934-20, and incorporating an amplification coefficient of 2.333′, this paralleled the recommended sampling ratio, encompassing a 300 mL test specimen situated within a 400 mL beaker, achieving an insulating depth of approximately 75 mm.
3. The temperature of the oven was meticulously calibrated to register 115 ± 1 °C, followed by a pre-heating intermission spanning 120 s.
4. Flasks, bearing labels from S2 through S18, were systematically introduced into the oven. Established sampling intervals were maintained. As a precautionary measure, protective hand gear was employed to mitigate thermal injuries. A structured aging schedule was punctiliously updated, reflecting the progressive removal of samples.
5. Upon conclusion of the heating cycle, samples were permitted an adequate cooling period, reverting to ambient conditions.
6. The contents housed within flasks S2 to S18 were systematically transferred to their respective designated containers (see Figure 1). It was imperative to ensure that these containers remained shielded from direct solar exposure.
7. A thorough cleaning regimen was implemented for flasks S2 through S18, involving washing, rinsing, and subsequent drying.

### 3.2. Refractive Index Measurement

The RI is a significant property that varies in proportion with the aging of TO and provides an insight into the optical characteristics of various instruments [21,23]. In the studies of [27], the sensitivity of a fiber optic sensor was mathematically expressed as a ratio of the transduced output voltage to the RI unit of the samples.

This can be mathematically expressed as follows:

(4) $$S=\frac{V_{out}}{n}\left[\frac{Volts}{RIU}\right]$$

where S represents the sensitivity of the optical fiber sensor, $V_{out}$ represents the fiber optic transduced output voltage, and n represents the RI of the test samples.

The apparatus used include:

1. Fresh and aged rapeseed ester oil;
2. Bellingham + Stanley refractometer;
3. Pipette;
4. Clean, lint-free cloth;
5. Distilled water;
6. Fisher Scientific ethanol (99%+).

**Figure 2:**
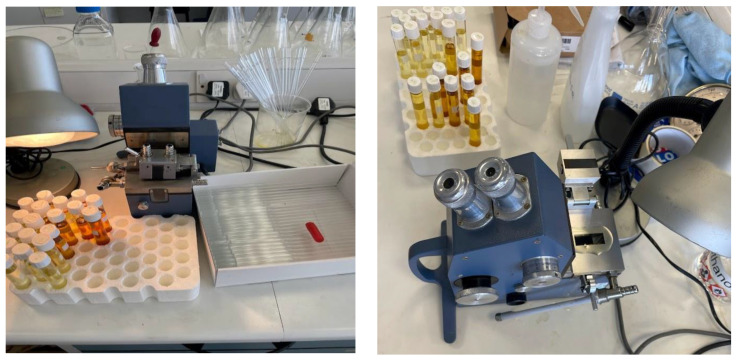
RI test bench.

The procedural steps were as follows:

1. The refractometer was adjusted to a RI value of 1.3333 utilizing distilled water for calibration purposes;
2. Ethanol, applied with a lint-free cloth, was used to meticulously cleanse the prism of the refractometer;
3. A fresh oil specimen was dispensed onto the prism using a pipette;
4. By closing the refractometer’s lid, the oil was uniformly distributed across the prism’s surface (see Figure 2);
5. The RI was ascertained by examining it through the device’s eyepiece;
6. The process from steps 2 through 5 was reiterated for samples that had undergone aging;
7. All recorded RI values were methodically logged, followed by a thorough cleaning of the refractometer and the testing area.

### 3.3. Fiber Optic Instrumentation Setup and Data Acquisition System

**Figure 3:**
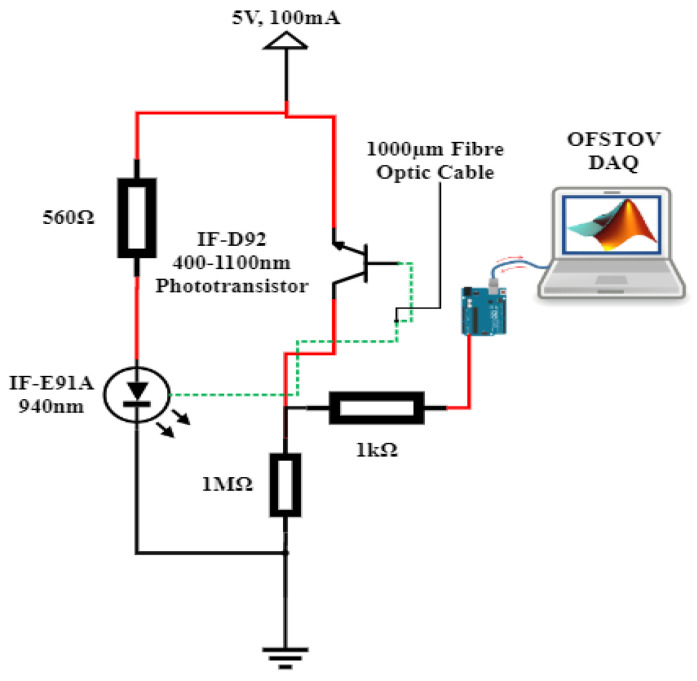
Opto-electronic configuration.

The fiber optic instrumentation apparatus, as delineated in Figure 3, encompasses a step-index polymethyl methacrylate plastic optical fiber (POF), coupled with an optical source, a detector, connector locking nuts, a sample holder, and a data acquisition (DAQ) system.

The designated POF comprises the core and the cladding. It is distinguished by its 1 mm diameter (diameter of core and cladding), 0.98 core diameter, 0.02 mm cladding thickness, a core RI of 1.492, and a cladding RI of 1.41. Through the utilization of a mechanical stripper, sensing lengths of 1.5 cm, 2 cm, and 3 cm were meticulously crafted. These sensing regions were subsequently integrated into both pristine and aged natural ester oil samples, adopting a U-shaped configuration. Notably, this U-shaped configuration has been demonstrated to exhibit superior sensitivity in comparison to the linear fiber orientation [28]. For the purpose of securing a stable interface between the optical source, the detector, and the optical fiber to the breadboard, connector locking nuts were used cautiously.

**Figure 4:**
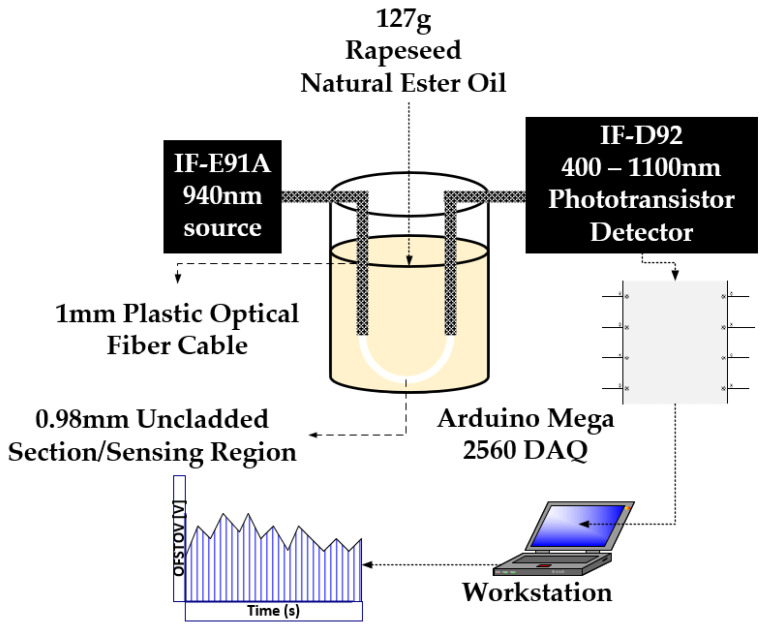
U-Shaped sensing configuration.

The optical sources consist of a 940 nm peak wavelength IF-E91A Fiber Optic Infrared LED, 470 nm peak wavelength IF-E92B Fiber Optic Blue LED, 530 nm peak wavelength IF-E93 Fiber Optic Green LED, and 645 nm peak wavelength IF-E96 Fiber Optic Red LED. The optical fiber detector is a highly sensitive NPN phototransistor (IF-D92, from Industrial Fiber Optics Inc., Tempe, AZ, USA). The detector spectral bandwidth ranges from 400 nm to 1000 nm and is capable of detecting optical signal from all four sources. The sample holder is an optically insulated container capable of holding up to 127 g of the samples (Figure 4).

The microcontroller utilized in this study is the Arduino Mega 2560 (Arduino Company, Ivrea, Italy), responsible for capturing the transduced analog voltage from the phototransistor via its analog input pin (A7). The Arduino microcontroller established a connection with the PC workstation through a USB port, which supplied a peak voltage of 5V DC to the power rails of the Arduino Mega 2560. This regulated voltage was then employed to energize the optical LEDs via current-limiting resistors (refer to Figure 3). With the integration of the MATLAB Support Library for Arduino Hardware, an effortless interface was established between the Arduino Hardware and the PC workstation equipped with MATLAB software R2021a. Data acquisition occurred in real time, with the data being showcased and archived for subsequent analysis within the MATLAB Simulink Software R2021a environment (Figure 4).

The apparatus included the following:

1. Arduino Mega 2560 microcontroller;
2. Light-emitting diodes (LED) (infrared, red, blue, and green);
3. Optically insulated fabricated vessel for oil;
4. 1000 µm uncladded plastic fiber optic cable;
5. Phototransistor;
6. Resistors;
7. PC workstation + MATLAB software R2021a;
8. Connecting cables;
9. Fisher Scientific isopropyl alcohol (99.5+%).

The procedural steps were as follows:

1. The opto-electronic setup was completed, as illustrated in Figure 3.
2. The sensing area of the optical fiber sensor was cleaned with isopropyl alcohol to eliminate any contaminants or residual traces of TO.
3. The optical fiber sensor underwent calibration, utilizing air as the reference fluid. The resulting transduced output voltage, as shown in Table 1, served as the calibration fluid for deviation adjustments during measurements of both fresh and aged samples. The setup was calibrated to the reference voltage in Table 1 before introducing each sample, ensuring that the results accurately reflected the sample characteristics.
4. Then, 127 g of the fresh oil sample was poured into the optically insulated fabricated vessel.
5. A settling time of one minute was observed before the transduced output voltage time series data were retrieved from MATLAB Simulink R2021a.
6. Steps 2 to 5 were repeated for the aged samples.
7. The voltage trend data for all samples were recorded and saved for subsequent analysis.

## 4. Results

### 4.1. Refractive Index Characterization

**Figure 5:**
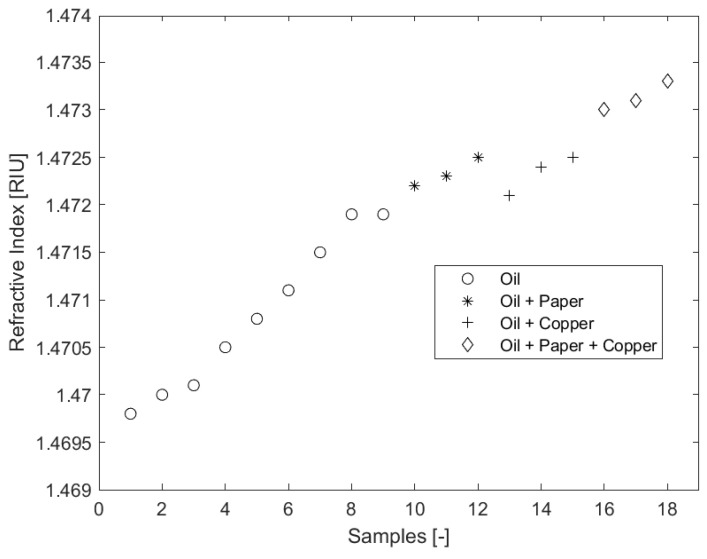
RI characterization with aging trend.

The RI characterization trend is shown in Figure 5. The plot shows a positive correlative behavior between RI and the aged samples for all samples. The influence of paper and copper on the RI property is also clearly shown in Figure 5. When a trend regression fit is inserted for each of the sample class, the intercept represents the offset or impact of the addition of paper/copper in the aging process. The influence of paper addition in accelerating the aging process is seen in the change in the RI offset value of $1.3\times 10^{-3}\left[RIU\right]$ for samples 1–9 and samples 10–12. Copper has a comparatively lower influence of $1\times 10^{-4}\left[RIU\right]$ for samples 1–9 and samples 13 to 15. The offset for samples 1–9 and samples 16–18 is $1.2\times 10^{-3}\left[RIU\right]$, a little lower than samples 10–12 with $1\times 10^{-4}\left[RIU\right]$ because of the influence of copper addition, and higher than samples 13–15 with $1.1\times 10^{-3}\left[RIU\right]$.

### 4.2. Sensitivity Analysis

In sensor and instrumentation studies, sensitivity analysis methodically evaluates how input variations impact the slope of measurement. This technique identifies key inputs affecting output, aiding in sensor selection, calibration, and performance troubleshooting. Essentially, sensitivity analysis is vital for understanding and enhancing sensor responsiveness to different conditions or inputs [32]. The key variables under consideration in this study are the impacts of the optical sources wavelengths, noise response analysis, the impact of sensing lengths, and the repeatability of results.

#### 4.2.1. Impact of Optical Source Wavelengths

**Figure 6:**
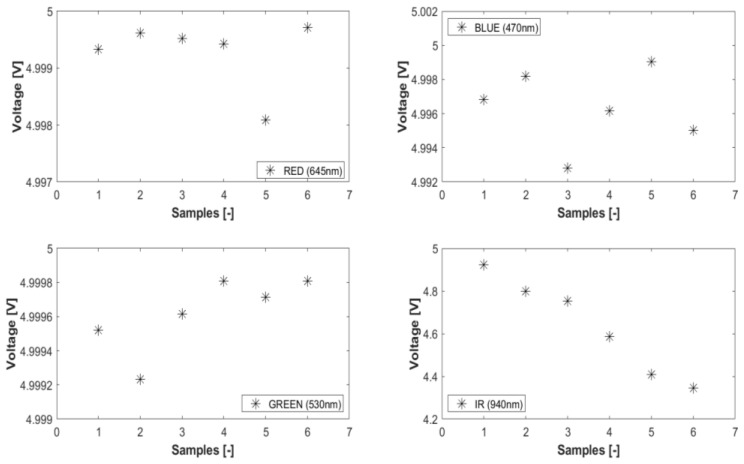
Trend data from varying optical wavelengths.

Under conditions of constant source power, fixed fiber orientation, and a uniform sensing length of 1.5 cm, data were gathered and averaged across various source inputs featuring different wavelengths for all the samples, as illustrated in Figure 6. One-third of the entire sample sets was utilized in this process. A negative linear trend was anticipated to rationalize the power loss on the cladding, attributable to the absorption within the evanescent field, as well as the corresponding increase in the RI, as delineated in Figure 5. The results obtained from the infrared optical source clearly demonstrate this anticipated response. Specifically, the infrared LED source trend has a negative linear correlation with the progression of sample aging, yielding a linear correlation coefficient value of 97.71%. Subsequent to the infrared LED, the red LED displays a faint negative linear correlation with sample aging, marked by a correlation coefficient value of 6%. Conversely, the green LED reveals a more pronounced positive correlation, a behavior that contravenes the prevailing theoretical understanding. The blue LED exhibits the most attenuated negative correlation among the observed trends.

The observations delineated in Figure 6 can be elucidated further by recalling the principle that optical frequency possesses an inverse relationship with wavelength. This relationship can be mathematically expressed as follows:

(5) c=fλ

where c represents the speed of the wave, f represents the frequency, and λ represents the wavelength of the wave. As per Planck’s quantum theory, this is mathematically expressed as follows:

(6) E=hf

where E represents the energy of the photon, h represents the Planck’s constant, which is approximately $6.626\times 10^{-34}$ joule-seconds, and f represents the frequency of the photon; the photon energy is proportional to the frequency of the photon. When combining Equations (5) and (6), the higher the optical wavelength, the lower the energy. As the infrared LED has the lowest energy, the absorbance will be higher [28], leading to a decreased intensity at the detector end.

#### 4.2.2. Noise Response Analysis

**Figure 7:**
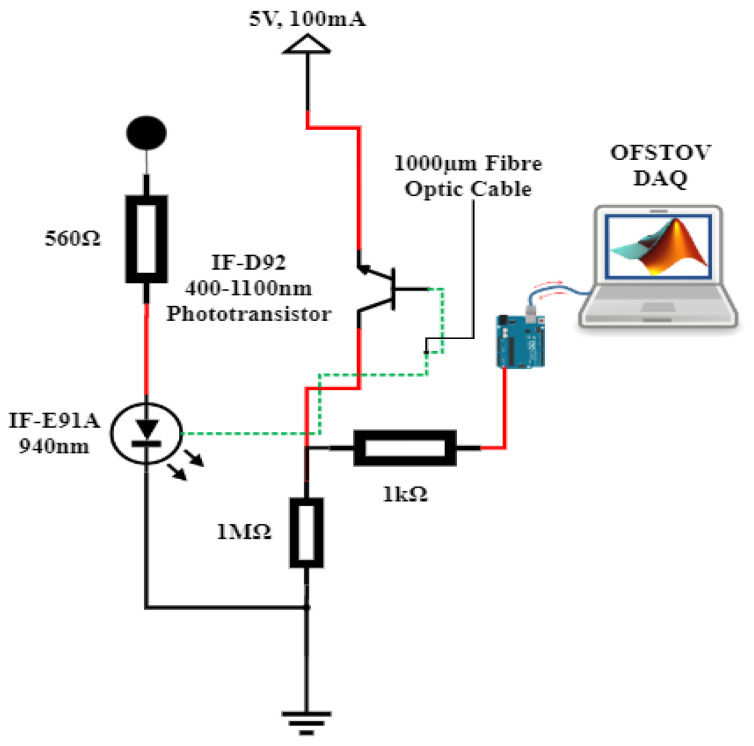
Opto-electronic configuration for noise analysis.

**Figure 8:**
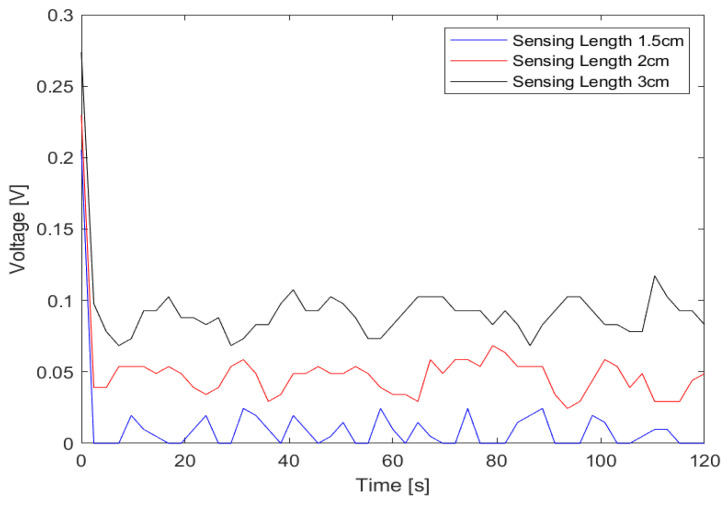
Noise analysis (time series plot).

**Figure 9:**
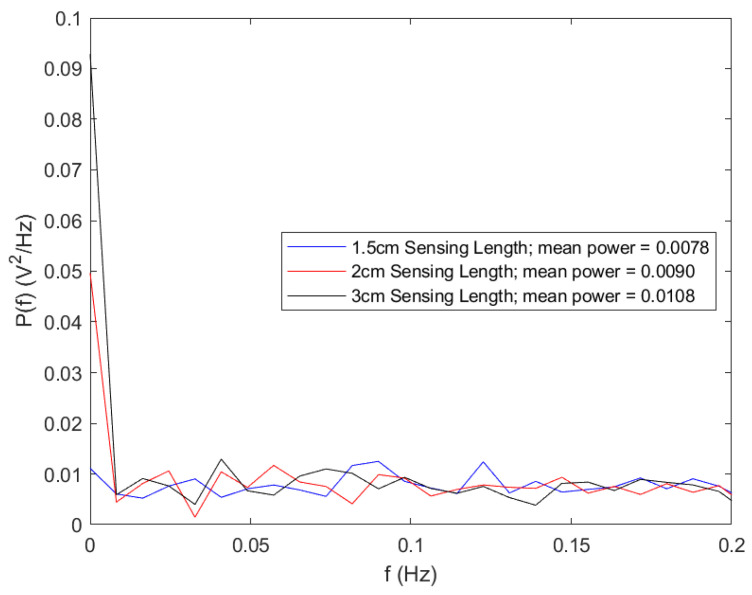
Noise analysis (power spectrum plot).

The conducted noise response analysis aimed to evaluate the resilience of the sensors (1.5 cm, 2 cm, and 3 cm) against noise interference. For this assessment, the source voltage to the LEDs was intentionally disconnected, as detailed in Figure 7. Subsequently, the detector indicated the ambient noise levels as captured by the various optical fiber sensors when they were exposed to air. The visualization of time series data and the power spectrum plot was facilitated using MATLAB Live Editor, as depicted in Figure 8 and Figure 9.

Regarding noise amplitude, the sensor featuring a 1.5 cm sensing length exhibited the most minimal noise amplitude, followed by the 2 cm sensor. The 3 cm sensor demonstrated the highest amplitude based on the time series data. This observation aligns with the noise power spectrum plot with the 1.5 cm sensor showing the least mean power value, followed by the 2 cm sensor and the 3 cm sensor. An examination of the noise peaks from the time series data further indicated that the 1.5 cm sensor detected the fewest peaks.

When considering noise resilience, the 1.5 cm sensor consistently demonstrated superior performance in mitigating noise interference.

#### 4.2.3. Impact of Sensing Lengths

Under consistent parameters, including stable source power, invariant fiber orientation, and a set source wavelength of 940 nm (as derived from the IF-E91A Fiber Optic Infrared LED, Industrial Fiber Optics, Inc., Tempe, AZ, USA), an examination was undertaken to understand the implications of different sensing lengths. As delineated in Section 4.2.1, the infrared LED demonstrated optimal performance in alignment with the foundational theoretical framework. As a result, it is favored for this analysis over alternative visible LED sources.

**Figure 10:**
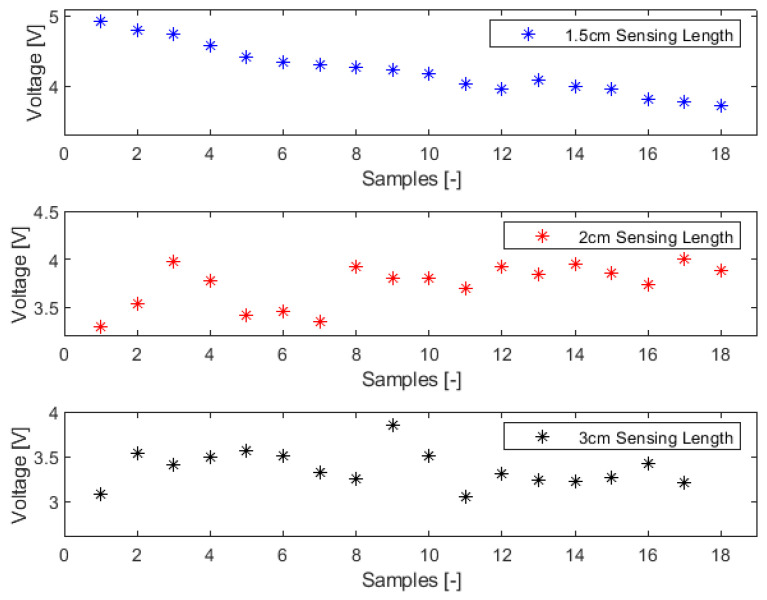
Aging trends based on sensing lengths.

As illustrated in Figure 10, the sensor’s response is influenced by variations in sensing lengths. The effect of noise, as elaborated upon in Section 4.2.2, is evident in the exhibited aging patterns. The observed sinusoidal noise trend, as shown in Figure 8, is mirrored in Figure 10. As the sensing length is extended, the influence of noise becomes increasingly discernible. Additionally, the longer the sensing length, the more prone the sensing length is to misalignment. The sensor with a 1.5 cm sensing length boasts a linear correlation coefficient of 94.56%, which is followed by the 2 cm sensor with a coefficient of 35.65%, and subsequently, the 3 cm sensor registering a value of 0.5%. Notably, the optical fiber with a sensing length of 1.5 cm displays optimal linearity, characterized by its descending trend. This trend provides an intuitive representation of the aging phenomenon and resonates with the instrumentation principles outlined in Section 2.

### 4.3. Repeatability of Results

Repeatability pertains to the degree of agreement among consecutive output readings when a consistent input is repetitively administered over a short time interval. This is achieved by maintaining uniform measurement conditions, utilizing the same instrument and observer, and keeping the location and other conditions consistent. As a metric of instrument precision, repeatability serves as an essential index of the reliability of measurement data. For measurement sensors, a high degree of precision in data reporting is not merely an expectation but often a stringent requirement [22].

In this study, repeatability is discussed in terms of the standard deviation of the transduced output voltage in the optical fiber sensor for seven random sample sets, each comprising fifty-one transduced output voltage values at a sampling interval of 2.4 s.

This can be mathematically expressed as follows:

(7) $$\sigma =\sqrt{\frac{1}{n-1}\sum_{i=1}^{n}{\left(V_{i}-\bar{V}\right)}^{2}}$$

where σ is the standard deviation, n represents the number of data points for each sample, and $\bar{V}$ represents the mean of the transduced voltage in the optical fiber sensor for each sample output voltage, $V_{i}$. For repeatability quoted in terms of standard deviation, the smaller the standard deviation, the higher the repeatability. High repeatability can be visualized from the normal distribution curve, where the peak is representative of the mean, and the spread of the distribution curve is representative of the standard deviation or repeatability. A narrower distribution curve would indicate the desired higher repeatability.

**Figure 11:**
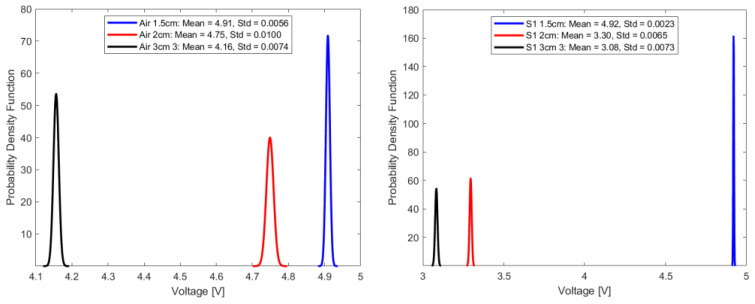
Repeatability plot of sensor in air and sample 1.

**Figure 12:**
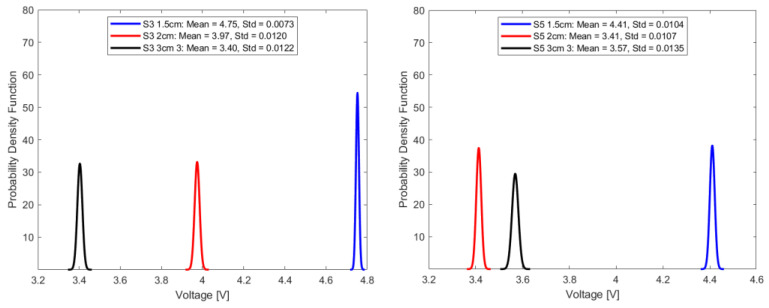
Repeatability plot of sensors in samples S3 and S5.

**Figure 13:**
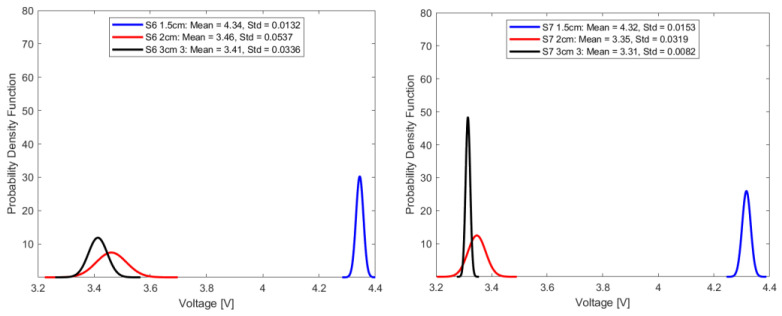
Repeatability plot of sensor in samples S6 and S7.

**Figure 14:**
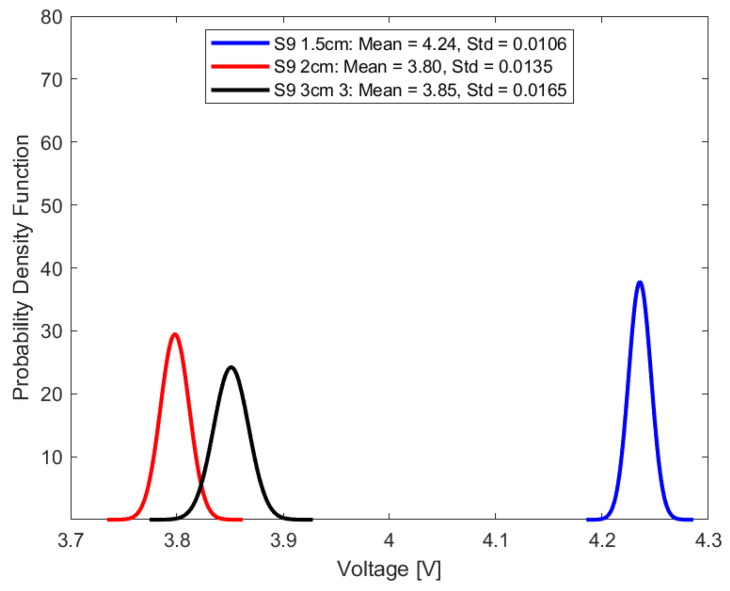
Repeatability plot of sensor in sample S9.

Mathematically, the Gaussian distribution function is expressed as:

(8) $$f\left(v\right)=\frac{1}{\sigma \sqrt{2\pi }}e^{-\frac{{\left(v-\bar{v}\right)}^{2}}{2\sigma^{2}}}$$

where f(v) is the probability density function for a given voltage value, v represents the voltage, and σ represents the standard deviation. Under consistent parameters, including stable source power, invariant fiber orientation, and a set source wavelength of 940 nm, Figure 11, Figure 12, Figure 13 and Figure 14 summarize the repeatability results.

In a comparative analysis utilizing standard deviation as a metric for repeatability, the optical fiber sensor with a 1.5 cm sensing length consistently outperformed its counterparts with longer sensing lengths. This notable distinction in repeatability was consistent across diverse conditions, including ambient air and select aged samples. Given its demonstrated high repeatability, the 1.5 cm optical fiber sensor is a more suitable choice for precision-based measurements and advanced instrumentation tasks, compared to the other longer two sensing lengths.

## 5. Discussion, Conclusions and Future Studies

In recent decades, the deployment of connected digital technologies (sensors and communication systems) in electrical substations has become an important driving force for the digitalization of electrical networks. Sensor deployment is essential in paving the way to digital twining. Continuous monitoring, particularly for oil-filled transformers, is essential due to potential oil deterioration and resultant aging by-products. While various methods exist to characterize TO, non-destructive online techniques are preferred. The rising emphasis is on fiber-optic sensors for detecting TO aging, given their resistance to electromagnetic interference and heightened sensitivity. These fibers are differentiated by factors like material and light propagation, each with unique benefits. Key elements of an optical fiber sensor system encompass the transmitter, sensing area, and detector, with intrinsic sensors being predominantly used.

In alignment with the principles of intensity-modulated fiber optic sensors and adhering to established industrial aging standards, eighteen samples of rapeseed natural ester oil underwent aging and were subsequently characterized utilizing a refractometer. The results from the RI exhibited a direct linear correlation with the duration of sample aging. Given the implications of alterations in the RI and the evanescent field on the optical fiber sensor, an inverse linear relationship was anticipated and confirmed across several optical sources. Notably, the infrared optical source had the most pronounced negative linear correlation, attributed to its reduced optical energy, saturation immunity, and consequently, elevated absorbance during sample traversal.

Notably, extending the sensing lengths increased the sensor’s sensitivity to environmental disturbances. Experimental data emphasize that sensors with the shortest sensing lengths exhibited optimal resistance to such environmental perturbations. As a result, the linearity of the optical fiber sensor is intrinsically linked to the length of the sensing region due to potential interference from noise. Additionally, the longer the sensing length, the more prone the sensing length is to misalignment. Consequently, sensors with minimized sensing lengths had superior linear outcomes, as evidenced by the correlation coefficient of determination. Further evaluations of repeatability, gauged by the standard deviation, reinforced the superiority of these short-length sensors in terms of repeatability, positioning them as favorable options for precise instrumentation compared to their counterparts with elongated sensing lengths.

Future research trajectories could delve into avenues for enhancing optical fiber sensitivity, including the use of filters and differential mode configuration for noise mitigation, the impact of incorporating coatings, and the effect of temperature changes on sensitivity response. Further investigations might scrutinize the repercussions of diverse intrusive configurations and choice of fiber optic material on sensitivity response. The potential integration of superhydrophobic coatings, aiming to curtail the influence of aging by-products on sensor sensitivity, also warrants further exploration. Complementary research endeavors could also focus on correlating fiber optic voltage measurements with conventional offline characterization methodologies, fostering the developments and implementation of online aging models.

## Figures and Tables

**Table 1:** Calibration voltage.

Sensing Lengths (cm)	LEDs Calibration Voltage (V)			
	Green	Red	Blue	Infrared
1.5	4.99	4.99	4.99	4.9093
2.0	4.99	4.99	4.99	4.7488
3.0	4.99	4.99	4.99	4.1563

## Data Availability

Data are contained within the article.

## Abbreviations

ABP	Aging by-products
ASTM	American Society for Testing and Materials
DAQ	Data acquisition system
LED	Light-emitting diode
PMMA	Polymethyl methacrylic
POF	Plastic optical fiber
RI	Refractive index
SOH	State of health
TO	Transformer oil

## References

[1] Elele U., Nekahi A., Arshad A., Fofana I. (2022). Towards Online Ageing Detection in Transformer Oil: A Review. Sensors.

[2] Hadjadj Y., Fofana I., Sabau J., Briosso E. (2015). Assessing insulating oil degradation by means of turbidity and UV/VIS spectrophotometry measurements. IEEE Trans. Dielectr. Electr. Insul..

[3] Alshehawy A.M., Mansour D.-E.A., Ghali M., Lehtonen M., Darwish M.M.F. (2021). Photoluminescence Spectroscopy Measurements for Effective Condition Assessment of Transformer Insulating Oil. Processes.

[4] Sangineni R., Nayak S.K., Becerra M. (2022). A Non-Intrusive and Non-Destructive Technique for Condition Assessment of Transformer Liquid Insulation. IEEE Trans. Dielectr. Electr. Insul..

[5] Rao U.M., Fofana I., Betie A., Senoussaoui M.L., Brahami M., Briosso E. (2019). Condition monitoring of in-service oil-filled transformers: Case studies and experience. IEEE Electr. Insul. Mag..

[6] Elele U., Fofana I., Nekahi A., McAulay K., Arshad A. Towards Intrusive Non-Destructive Online Ageing Detection of Transformer Oil Leveraging Bootsrapped Machine Learning Models. Proceedings of the 2023 IEEE Electrical Insulation Conference (EIC).

[7] Siva Sai R., Rafi J., Farook S., Kumar N., Parthasarathy M., Ashok Bakkiyaraj R. (2020). Degradation studies of electrical, physical and chemical properties of aged transformer oil. J. Phys..

[8] Alshehawy A.M., Mansour D.-E.A., Ghali M., Rezk A. Evaluating the impact of aging in field transformer oil using optical spectroscopy techniques. Proceedings of the 2017 IEEE 19th International Conference on Dielectric Liquids (ICDL).

[9] Perrier C., Marugan M., Beroual A. (2012). DGA comparison between ester and mineral oils. IEEE Trans. Dielectr. Electr. Insul..

[10] Mansour D.-E.A. A new graphical technique for the interpretation of dissolved gas analysis in power transformers. Proceedings of the 2012 Annual Report Conference on Electrical Insulation and Dielectric Phenomena.

[11] N’cho J.S., Fofana I. (2020). Review of Fiber Optic Diagnostic Techniques for Power Transformers. Energies.

[12] Alshehawy A.M., Mansour D.A., Ghali M. Condition Assessment of Aged Transformer Oil Using Photoluminescence-Based Features. Proceedings of the 2021 IEEE 5th International Conference on Condition Assessment Techniques in Electrical Systems (CATCON).

[13] Fofana I., Borsi H., Gockenbach E., Farzaneh M. (2007). Aging of transformer insulating materials under selective conditions. Eur. Trans. Electr. Power.

[14] (2018). British Standard: Insulating Liquids. Test Methods for the Determination of Interfacial Tension of Insulating Liquids. Determination with the Ring Method.

[15] Jin L., Kim D., Abu-Siada A., Kumar S. (2022). Oil-Immersed Power Transformer Condition Monitoring Methodologies: A Review. Energies.

[16] Rahman O., Islam T., Ahmad A., Parveen S., Khera N., Khan S.A. (2021). Cross Capacitance Sensor for Insulation Oil Testing. IEEE Sens. J..

[17] Rahman O., Islam T., Khera N., Khan S.A. (2021). A Novel Application of the Cross-Capacitive Sensor in Real-Time Condition Monitoring of Transformer Oil. IEEE Trans. Instrum. Meas..

[18] Mahanta D.K., Laskar S. (2018). Water Quantity-Based Quality Measurement of Transformer Oil Using Polymer Optical Fiber as Sensor. IEEE Sens. J..

[19] Maria de Fátima F.D., Radwan A. (2017). Optical fiber sensors for IoT and Smart Devices.

[20] Riziotis C., El Sachat A., Markos C., Velanas P., Meristoudi A., Papadopoulos A. Assessment of fiber optic sensors for aging monitoring of industrial liquid coolants. Proceedings of the SPIE OPTO.

[21] Teng C., Min R., Zheng J., Deng S., Li M., Hou L., Yuan L. (2021). Intensity-Modulated Polymer Optical Fiber-Based Refractive Index Sensor: A Review. Sensors.

[22] Morris A.S., Langari R. (2021). Measurement and Instrumentation.

[23] Terdale J., Ghosh A. (2022). An intensity-modulated optical fiber sensor with agarose coating for measurement of refractive index. Int. J. Syst. Assur. Eng. Manag..

[24] Elele U., Nekahi A., Arshad A., McAulay K., Fofana I. (2023). Sensitivity Analysis of Intensity-Modulated Plastic Optical Fiber Sensors for Effective Ageing Detection in Rapeseed Transformer Oil. Preprints.

[25] Pligovka A., Lazavenka A., Turavets U., Hoha A., Salerno M. (2023). Two-Level 3D Column-like Nanofilms with Hexagonally–Packed Tantalum Fabricated via Anodizing of Al/Nb and Al/Ta Layers—A Potential Nano-Optical Biosensor. Materials.

[26] Pligovka A. (2021). Reflectant Photonic Crystals Produced via Porous-Alumina-Assisted-Anodizing OF Al/Nb AND Al/Ta SYSTEMS. Surf. Rev. Lett..

[27] Hayber E., Tabaru T.E., Güçyetmez M. (2021). Evanescent Field Absorption-Based Fiber Optic Sensor for Detecting Power Transformer Oil Degradation. Fiber Integr. Opt..

[28] Patil J.J., Ghosh A. Intensity Modulation based U shaped Plastic Optical Fiber Refractive Index Sensor. Proceedings of the 2022 6th International Conference on Trends in Electronics and Informatics (ICOEI).

[29] Khijwania S.K., Srinivasan K.L., Singh J.P. (2005). An evanescent-wave optical fiber relative humidity sensor with enhanced sensitivity. Sens. Actuators B Chem..

[30] Ghosh D., Khastgir D. (2018). Degradation and Stability of Polymeric High-Voltage Insulators and Prediction of Their Service Life through Environmental and Accelerated Aging Processes. ACS Omega.

[31] (2012). Standard Test Method for Oxidative Aging of Electrical Insulating Petroleum Oils by Open-Beaker Method.

[32] Iooss B., Saltelli A., Ghanem R., Higdon D., Owhadi H. (2016). Introduction to Sensitivity Analysis. Handbook of Uncertainty Quantification.

